# Loss of niche-satellite cell interactions in syndecan-3 null mice alters muscle progenitor cell homeostasis improving muscle regeneration

**DOI:** 10.1186/s13395-016-0104-8

**Published:** 2016-10-04

**Authors:** Addolorata Pisconti, Glen B. Banks, Farshad Babaeijandaghi, Nicole Dalla Betta, Fabio M. V. Rossi, Jeffrey S. Chamberlain, Bradley B. Olwin

**Affiliations:** 1 Department of Cellular, Molecular and Developmental Biology, University of Colorado at Boulder, Boulder, CO 80309 USA; 2 Department of Biochemistry, Institute of Integrative Biology, University of Liverpool, Liverpool, L69 7ZB UK; 3 Department of Neurology, University of Washington, Mail Stop 357720, Seattle, WA 98195 USA; 4 The Biomedical Research Centre, UBC, Vancouver, BC V6T 1Z Canada

**Keywords:** Satellite cells, Muscle regeneration, Muscular dystrophy, Niche, Cell adhesion, Cell migration, Syndecan-3, Pax7

## Abstract

**Background:**

The skeletal muscle stem cell niche provides an environment that maintains quiescent satellite cells, required for skeletal muscle homeostasis and regeneration. Syndecan-3, a transmembrane proteoglycan expressed in satellite cells, supports communication with the niche, providing cell interactions and signals to maintain quiescent satellite cells.

**Results:**

Syndecan-3 ablation unexpectedly improves regeneration in repeatedly injured muscle and in dystrophic mice, accompanied by the persistence of sublaminar and interstitial, proliferating myoblasts. Additionally, muscle aging is improved in syndecan-3 null mice. Since syndecan-3 null myofiber-associated satellite cells downregulate Pax7 and migrate away from the niche more readily than wild type cells, syxndecan-3 appears to regulate satellite cell homeostasis and satellite cell homing to the niche.

**Conclusions:**

Manipulating syndecan-3 provides a promising target for development of therapies to enhance muscle regeneration in muscular dystrophies and in aged muscle.

**Electronic supplementary material:**

The online version of this article (doi:10.1186/s13395-016-0104-8) contains supplementary material, which is available to authorized users.

## Background

Muscular dystrophy is a family of genetic disorders characterized by progressive muscle loss, chronic inflammation and replacement of muscle tissue with fibrotic tissue [1, 2]. In several types of muscular dystrophy, the continuous myofiber damage caused by the primary genetic defect imposes a high demand for myofiber repair, which is sustained by muscle progenitors. The proliferative potential of the resident muscle progenitors, named satellite cells, is presumed to be prematurely exhausted in muscular dystrophy, abrogating muscle regeneration and leading to fibrosis [3–7]. Although a thorough understanding of the molecular mechanisms regulating satellite cells in muscular dystrophy is incomplete, cell-intrinsic mechanisms, such as telomerase expression [8], cell cycle regulators [9], and cell-intrinsic disruptions of self-renewal and cell division upon dystrophin loss from satellite cells [10] as well as non cell autonomous regulation, including the extracellular environment [11–14], play critical roles in regulating satellite cell homeostasis.

During aging, myofiber size progressively decreases with an accompanying loss of fast twitch myofibers, leading to reduced overall muscle mass and strength that, when severe, results in sarcopenia. Loss of muscle mass and strength is accompanied by increased matrix deposition (fibrosis) and increased fat infiltration. Skeletal muscle regeneration is impaired in aged muscle and associated with cell-intrinsic deficits in satellite cell function [15–20]; however, satellite cell contribution to sarcopenia has been recently questioned, although a contribution of satellite cell loss to aging-associated fibrosis is supported [21].

Satellite cells in G0 phase reside within the musculature and are poised to rapidly activate in response to injury [22–26]. Upon activation, satellite cells re-enter the cell cycle, migrate away from their niche, and proliferate as myoblasts, eventually undergoing terminal differentiation into myocytes that fuse into pre-existing damaged muscle fibers or fuse to one another generating new muscle fibers [27]. During regeneration, a portion of satellite cells returns to its niche, re-enters quiescence, and expresses Pax7 but no other myogenic transcription factors [27–29]. The transmembrane heparan sulfate proteoglycan syndecan-3, a component of the satellite cell niche, controls satellite cell homeostasis by regulating signaling pathways within the niche [12, 14, 30–32]. Moreover, members of the Syndecan family regulate cell-cell adhesion and cell-matrix adhesion via interaction with integrins and cadherins [33]. Following a muscle injury, syndecan-3 null (*Sdc3* ^*−/−*^) satellite cells fail to replenish the resident pool of quiescent satellite cells within the niche [14] and therefore syndecan-3 appears to regulate satellite cell homeostasis [14].

We show that syndecan-3 loss alters satellite cell adhesion to the myofiber, altering interactions with the niche and (i) improves muscle regeneration upon repeated acute muscle injuries, (ii) rescues muscle histopathology and function in dystrophic muscle tissue, and (iii) improves muscle aging with a reduction in fibrosis. The lifelong improvement in muscle regeneration observed in *Sdc3* ^*−/−*^ muscle arises in part by altered satellite cell homeostasis and changes in satellite cell adhesiveness to the myofiber.

## Methods

### Mice

Mice were housed in a pathogen-free facility at the University of Colorado at Boulder, USA, or at the University of Liverpool, UK. All injuries and other procedures were performed at the University of Colorado, and protocols were approved by the IACUC at the University of Colorado. Animals housed at the University of Liverpool were used in accordance with the Animals (Scientific Procedures) Act 1986 and the EU Directive 2010/63/EU and after local ethical review and approval by Liverpool University’s Animal Welfare and Ethical Review Body (AWERB). *Sdc3* ^*−/−*^ mice were donated by Dr. Heikki Rauvala, University of Helsinki, Finland. *Mdx* ^*4cv*^ mice were donated by Dr. Jeffrey Chamberlain, University of Washington, Seattle, USA. Generation of double mutant colonies is described in details in Additional file 1. In all experiments, wild type and *mdx* ^*4cv*^ *;Sdc3* ^*+/+*^ controls were all siblings or closely related, inbred, sex- and age-matched animals for all transgenic lines.

### Immunofluorescence

Tissue samples were collected and either immediately frozen in liquid nitrogen-cooled isopentane or fixed in 10 % formalin. For all immunofluorescence staining except Myf5 and Pax7, sections were fixed with 4 % paraformaldehyde (PFA) in phosphate buffered saline (PBS) for 10 min at room temperature. For Myf5 staining, sections were fixed for 10 min with acetone at −20 °C. For Pax7 staining, sections were either fixed and stained using an anti-Pax7 rabbit polyclonal antibody (Genetex) or non fixed, processed for antigen retrieval, and stained with an anti-Pax7 mouse monoclonal antibody (DSHB). The antibodies used were as follows: rabbit polyclonal anti-Pax7 (Genetex) at 1:250; rabbit polyclonal anti-laminin (Sigma) 1:150; rat polyclonal anti-laminin α2 (Sigma) 1:100; rat anti-F4/80 (Genetex) 1:200; rat anti-BrdU (Serotec) 1:100; mouse anti-Pax7 monoclonal (DSHB) 1:200; rabbit anti-myogenin (SCBT) 1:50; rabbit anti Myf5 (SCBT) 1:200; rat anti-CD31 (BD Biosciences) 1:100; rabbit anti-NG2 (Chemicon) 1:200; rabbit anti-Ki67 (Abcam) 1:400; rat anti-Sca1 (unconjugated, PE-conjugated, APC-Cy7-conjugated and FITC-conjugated were all from BD Biosciences), 1:100; rabbit anti-GFP (BD Biosciences), 1:400. Secondary antibodies conjugated with Alexa594, Alexa555, Alexa488, or Alexa647 (Molecular Probes) were used at 1:500 dilution. Vectashield with DAPI (Vector Laboratories) was used as a mounting medium.

### Sirius red staining

Flash-frozen sections were fixed for 1 h at 56 °C in Bouin’s fixative, washed in water, stained for 1 h in Master*Tech Picro Sirius Red, washed in 0.5 % acetic acid, dehydrated, equilibrated with xylene, and mounted using Permount™.

### Trichrome staining

Trichrome staining was performed according to standard protocols by Premier Laboratory LLC, Boulder, CO, on paraffin-embedded tissues fixed in 10 % formalin in neutral buffered saline and preserved in 70 % ethanol.

### Morphometric analysis

Myofiber cross-sectional area and numbers in uninjured and injured TA muscles were quantified as previously described [14]. The fibrotic index (% collagen + area in Sirius Red staining relative to total section area) was quantified by selecting red pixels in Adobe Photoshop, deleting all non-red pixels, converting the resulting image to a binary image, and counting red pixels using the ImageJ Analyze Particles function. The necrotic index was calculated by counting the number of mIgG+ myofibers and normalizing to total number of myofibers in the image. Capillary density was calculated by measuring the numbers of capillary around each fiber on alternate fibers in order to avoid overlapping scorings. Ten sections per mouse for three different mice were scored.

### Endurance training

Female and male mice of different genotypes were individually housed in cages equipped with a training wheel connected to a bicycle computer (Schwinn) with ad libitum access to food and water for 3 weeks. Time and distance run were recorded daily.

### Muscle physiology

Mice were anesthetized with 2,2,2-tribromoethanol (Sigma) such that they were insensitive to tactile stimuli. Peak isometric force of the TA muscle was analyzed in situ via nerve stimulation. First, we found the maximum force-producing capacity of each muscle at its optimum length according to maximal stimulation over 300 ms to elicit tetanic contraction. The peak force was then divided by the unit area of muscle to obtain specific force (kN/m^2^) using the equation: specific force = peak force × muscle length × 0.6 × 1.04/muscle weight [34]. Next, we measured protection from contraction-induced injury. The force-producing capacity of the muscle was measured immediately prior to increased length changes during maximal stimulation at 20-s intervals. Length changes were increased in 5 % increments from 5 to 45 % of muscle fiber length to produce injury. The rate of length change was 2 lengths/s.

### Western blotting

Quadriceps were homogenized in 20 mM HEPES, 50 mM KCl, 1 mM DTT, 2 mM MgCl_2_, 0.5 mM EDTA, 0.5 % NP40 supplemented with protease inhibitor cocktail (Complete, Roche), and phosphatase inhibitors (1 mM Na_3_VO_4_ + 1 mM NaF) using an UltraTurrex homogenizer followed by incubation on ice for 20 min and then cleared by centrifugation at 13,000 rpm for 10 min at 4 °C. Western blot was performed as previously described [14]. The antibodies used were as follows: rabbit polyclonal anti-dystrophin (Abcam) at 1:1000; rabbit polyclonal anti-utrophin (kindly donated by Dr. Froehner, University of Washington, Seattle) 1:2000. Anti-rabbit conjugated secondary antibodies (Santa Cruz) were used at 1:10,000, and HRP activity was visualized using the ECL plus system (Amersham).

### RT-PCR primers

Dystrophin forward: CAGCTGCAGAACAGGAGTT.

Dystrophin reverse: GCATCTACTGTGTGAGGACC.

### Mouse injury

Mice were anesthetized with isofluorane and the right TA muscle was injected with 50 μL of 1.2 % BaCl_2_ [35] in three places along the length of the muscle and then both the injured muscle and the contralateral uninjured muscle harvested at the indicated time points. For repeated injuries, the same TA muscle was injured as above for a total of three times with 3-week intervals between injuries. The injured TA muscle and the contralateral uninjured TA muscle were harvested 3 weeks after the last injury.

### Fluorescence-activated cell sorting

Hindlimb muscles of 3–6-month-old *Sdc3* ^*−/−*^ and littermate wild type female mice were dissected, minced, and digested in 400 U/mL collagenase type I in Ham’s F-12C (F12 + 0.4 mM CaCl_2_) at 3 °C for 1 h, gently vortexing every 10 min. Collagenase was diluted 1:3 with F12C + 15 % horse serum (HS) and tissue debris removed by centrifugation at 30×*g* for 5 min (pellet contains large debris) followed by straining of the supernatant (containing mononucleated cells and smaller debris) through 40-μm cell strainers (BD Falcon). Flow through was then centrifuged at 300×*g*, and the cell pellets were re-suspended in PBS + 5 % fetal bovine serum (FBS) and incubated for 45 min at 4 °C with 1:100 phycoerythrin (PE)- or fluorescein isothiocyanate (FITC)-directly conjugated rat anti-Sca1 antibody (BD Biosciences) and 1:500 chicken anti-Sdc4 [12] followed by an incubation for 45 min at 4 °C with Alexa 647-conjugated anti-chicken IgY (Molecular Probes). Sca1+, Sca1+/Sdc4−, and Sca1−/Sdc4+ cells were sorted on a MoFlo XDP Cell Sorter (Dako Cytomation) into Ham’s F12C + 15 % horse serum (HS) and cultured in a myogenic growth medium (F12C + 15 % HS + 2 nM FGF2) or transplanted, see transplantation details below. To assess the expression of Pax7, Pax3, and Myf5 and MyoD, fibro-adipogenic progenitors (FAPs) were sorted as Hoechst^mid^ PI^lo^ CD45^−^ CD31^−^ Sca1^+^ CD34^+^ cells and muscle progenitors (MPs) were sorted as Hoechst^mid^ PI^lo^ CD45^−^ CD31^−^ Sca1^−^ CD34^+^ as previously described [36] directly into lysis buffer (CellsDirect Resuspension & Lysis Buffer, Life Technologies).

### Droplet Digital PCR

Following RNA isolation (CellsDirect Resuspension & Lysis Buffer, Life Technologies) and reverse transcription (High Capacity cDNA Reverse Transcription Kit, Life Technologies) according to the manufacturer’s instructions, complementary DNA (cDNA) was diluted five times in TE buffer and 5 μL were used in a reaction mix containing Droplet Digital™ PCR Supermix (BioRad), 1× TaqMan probes from Life Technologies [Pax7 (Mm03053796-s1), Myf5 (Mm00435125-m1), Hprt (Mm00446968_m1), Pax3 (Mm00435493_m1), and Myod1 (Mm00440387_m1)] and H_2_O. Droplets were generated with a QX100 droplet generator (BioRad), after mixing 20 μL of reaction mix and 70 μL of droplet generator oil (BioRad). The emulsified samples were loaded onto 96-well plates, and endpoint PCRs were performed in C1000 Touch thermal cycler (BioRad) at the following cycling conditions (95 °C for 10 min, followed by 45 cycles of 94 °C for 30 s and 60 °C for 1 min, followed by 98 °C for 10 min). The droplets from each sample were read through the QX100 droplet reader (BioRad). Resulting PCR-positive and PCR-negative droplets were counted using QuantaSoft software (BioRad). Expression levels were normalized to Hprt.

### Cell transplantation

Sca1+ cells were FACS-isolated as described above from *Sdc3* ^*+/+*^ *;β-actin-GFP* and *Sdc3* ^*−/−*^ *;β-actin-GFP* mice, centrifuged, and washed twice with sterile 0.9 % NaCl to remove the serum, re-suspended into 0.9 % NaCl at the concentration of 2400 cells/μL and 30 μL (~70,000 cells) immediately injected into the right TA muscle of wild type mice which had been injured 4 h before with an injection of 30 μL of 1.2 % BaCl_2_. Three weeks after animals were sacrificed, the right (injured and transplanted) and left (uninjured, untransplanted) TA muscles were dissected and cryopreserved for subsequent histological analysis.

### Myofiber isolation and culture

The gastrocnemius muscles of wild type and *Sdc3* ^*−/−*^ mice were dissected and incubated with 400 U/mL collagenase type I in F12C at 37 °C, with gentle mixing by inversion every 15 min for 1 h 30 min, after which collagenase was diluted 1:5 with F12C + 15 % HS and muscles gently rocked at room temperature for 15 min to allow for myofiber release from the digested muscle. Individual myofibers were manually picked and transferred to fresh F12C + 15 % HS using a sterile, flame-polished Pasteur pipette. Myofibers were cultured in suspension in F12C + 15 % HS + 2 nM FGF2 in non-coated sterile petri dishes unless otherwise specified and transferred every 24 h to fresh medium.

### Microscopy, image processing, and figure preparation

Micrographs were taken with a Leica TCS SP2 AOBS confocal microscope using dedicated Leica software, or with a Nikon (Eclipse E800) epifluorescence microscope using Slidebook v4.1 acquisition software (Intelligent Imaging Innovations Inc.) coupled to a Cooke Sensicam digital camera or with an EVOS-FL inverted microscope (Life Technologies). Lenses used with the Leica confocal microscope were either HC PL APO 20×/0.70 IMM CORR CS or HCX PL APO 40×/1.25–0.75. Lenses used with the Nikon Eclipse microscope were Nikon Plan Fluor either 40×/0.75 DIC M or 20×/0.50 Ph1 DLL. Lenses used with the EVOS microscope were PL FL, either 10× LWD PH, 0.25NA/9.2WD or 40× LWD PH, 0.56NA/1.6WD. All digital microscopic images were acquired at room temperature. For figure preparation, images were exported in Adobe Photoshop, if necessary brightness and contrast adjusted and the background removed for the entire image, the image cropped and individual color channels extracted (when required) without color correction or gamma adjustments.

### Statistical analysis

To assess statistical significance, two-tailed, unpaired Student’s *t* test or one-way analysis of variance (ANOVA) were performed. *p* < 0.05 was considered significant. At least three different animals per genotype and per age group were used in all experiments. Cell culture experiments (both myofiber and myoblast cell cultures) were repeated three independent times using three different animals per genotype group. For force measurements, five to seven animals per genotype were used. For muscle function testing (voluntary wheel), three to seven animals per genotype group were used.

## Results

### Dystrophic mice lacking syndecan-3 show improved muscle histopathology and function

**Fig. 1:**
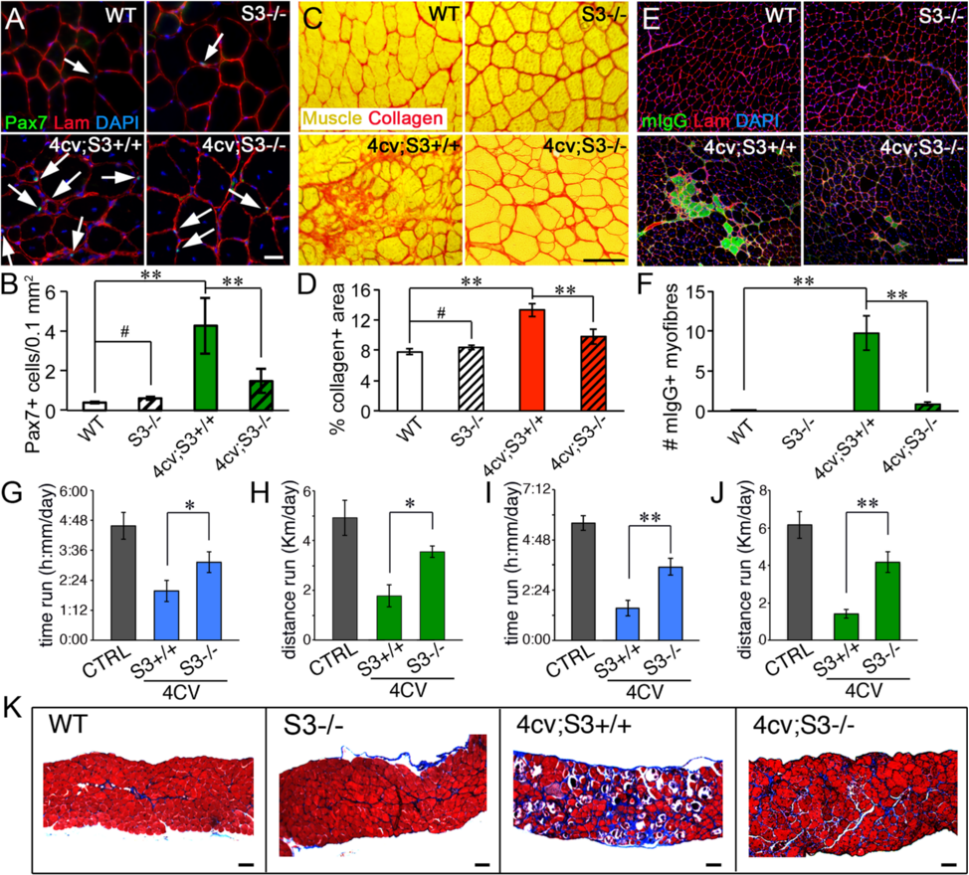
Loss of syndecan-3 improves dystrophic muscle histopathology and muscle function. **a**, **b** Pax7+ satellite cells are present in equal numbers in wild type and *Sdc3* ^*−/−*^ muscle (*top panels*) but are reduced in *mdx* ^*4cv*^ *;Sdc3* ^*−/−*^ (4cv;S3*−/−*) compared to *mdx* ^*4cv*^ *;Sdc3* ^*+/+*^ (4cv;S3+/+) muscles (*lower panels*). Interstitial Pax7 immunoreactive cells were occasionally observed in *mdx* ^*4cv*^ *;Sdc3* ^*−/−*^ muscle; these were rare and not scored. Average numbers of Pax7+ sublaminar cells plotted in **b** as percentage of total area. **c**–**f** Cross sections of wild type, *Sdc3* ^*−/−*^ (S3*−/−*), *mdx* ^*4cv*^ *;Sdc3* ^*+/+*^ (4cv;S3+/+), and *mdx* ^*4cv*^ *;Sdc3* ^*−/−*^ (4cv;S3*−/−*) mice were stained to detect collagen (**c**, *red*), muscle tissue (**c**, *yellow*), mouse immunoglobulins (**e**, *green*), and laminin (**e**, *red*). Connective tissue quantified as area stained in *red* (**c**) and plotted in **d** as a percentage of the total area. Myofibers with increased sarcolemmal permeability quantified as number of myofibers containing mouse IgG immunostaining in **e** and plotted in **f** as percentage of the total myofiber numbers. **g**–**j** Exercise performance in male (**g**, **h**) and female (**i**, **j**) *mdx* ^*4cv*^ *;Sdc3* ^*−/−*^ and *mdx* ^*4cv*^ *;Sdc3* ^*+/+*^ sex- and age-matched mice measured as time run (**g** and **i**) or distance run (**h** and **j**) during 3 weeks of volunteer running. Daily averages for each genotype are plotted. Non-dystrophic *Sdc3* ^*+/+*^ and *Sdc3* ^*−/−*^ mice were averaged and plotted as control (**g–j**). **k** Diaphragm histology in exercised mice by trichrome staining. *Error bars* are S.E.M. ** = *p <* 0.01, * = *p <* 0.05. *Scale bars* are 100 μm in **c**, 50 μm in **e**, and 30 μm in **a**

The numbers of satellite cells per myofiber is increased in *Sdc3* ^*−/−*^ mice [12]; however, the numbers of Pax7+ satellite cells in uninjured *Sdc3* ^*−/−*^ muscles are similar to those found in wild type muscles (Fig. 1a, top panels, b). Loss of Pax7+ satellite cells in *Sdc3* ^*−/−*^ muscle occurs after injury-induced regeneration [14] and thus we asked whether chronic injury would also lead to loss of Pax7+ satellite cells. *Sdc3* ^*−/−*^ mice were bred with dystrophic *mdx* ^*4cv*^ mice (Additional file 1: Figure S1A) to determine if syndecan-3 loss would exacerbate loss of Pax7+ satellite cells. We chose the *mdx* ^*4cv*^ strain [37] because it develops a more severe form of muscular dystrophy than *mdx* mice due to lower numbers of revertant muscle fibers [38]. As expected, a reduction in Pax7+ satellite cells was observed in *mdx* ^*4cv*^ *;Sdc3* ^*−/−*^ mice compared to *mdx* ^*4cv*^ *;Sdc3* ^*+/+*^ mice (Fig. 1a, b), but, surprisingly, the histopathology of *mdx* ^*4cv*^ *;Sdc3* ^*−/−*^ muscles was improved compared to *mdx* ^*4cv*^ *;Sdc3* ^+/+^ muscles (Fig. 1c–f). Fibrosis was reduced in *mdx* ^*4cv*^ *;Sdc3* ^*−/−*^ muscles (Fig. 1c, d), accompanied by a reduction in sarcolemmal permeability (Fig. 1e, f) compared to *mdx* ^*4cv*^ *;Sdc3* ^*+/+*^ littermate controls. These differences were not due to a strain effect since (i) *Sdc3* ^*−/−*^ mice and *mdx* ^*4cv*^ mice share the same background (C57Bl/6); (ii) all experiments were carried out using the inbred progeny of *Sdc3* ^*−/−*^ and *mdx* ^*4cv*^ founders (Additional file 1: Figure S1A).

Improved muscle function in syndecan-3 null dystrophic mice accompanied the improved muscle histopathology as compared to *mdx* ^*4cv*^ *;Sdc3* ^*+/+*^ mice. Both male and female *mdx* ^*4cv*^ *;Sdc3* ^*−/−*^ mice ran for longer distances and for longer periods of time than *mdx* ^*4cv*^ *;Sdc3* ^*+/+*^ controls when assayed on a voluntary wheel, performing similar to the times and distances recorded for wild type mice (Fig. 1g, j). This was not due to an intrinsically increased propensity of *Sdc3* ^*−/−*^ mice to perform better in endurance training tests as no significant differences in time and distance run were recorded for *Sdc3* ^*−/−*^ non-dystrophic mice compared to wild type mice. The diaphragm muscle, which was severely affected following voluntary running in *mdx* ^*4cv*^ *;Sdc3* ^*+/+*^ mice, was dramatically improved in *mdx* ^*4cv*^;*Sdc3* ^*−/−*^ mice following voluntary exercise (Fig. 1k). Amelioration of the dystrophic phenotype in *mdx* ^*4cv*^;*Sdc3* ^*−/−*^ mice was likely maintained throughout life as in 14-month-old (Additional file 1: Figure S1B, C) and 19-month-old (Additional file 1: Figure S1D, E) muscle, collagen deposition is reduced in *mdx* ^*4cv*^ *;Sdc3* ^*−/−*^ compared to *mdx* ^*4cv*^ *;Sdc3* ^*+/+*^ mice (Additional file 1: Figure S1B-E).

### Regeneration is improved in dystrophic muscle lacking syndecan-3

**Fig. 2:**
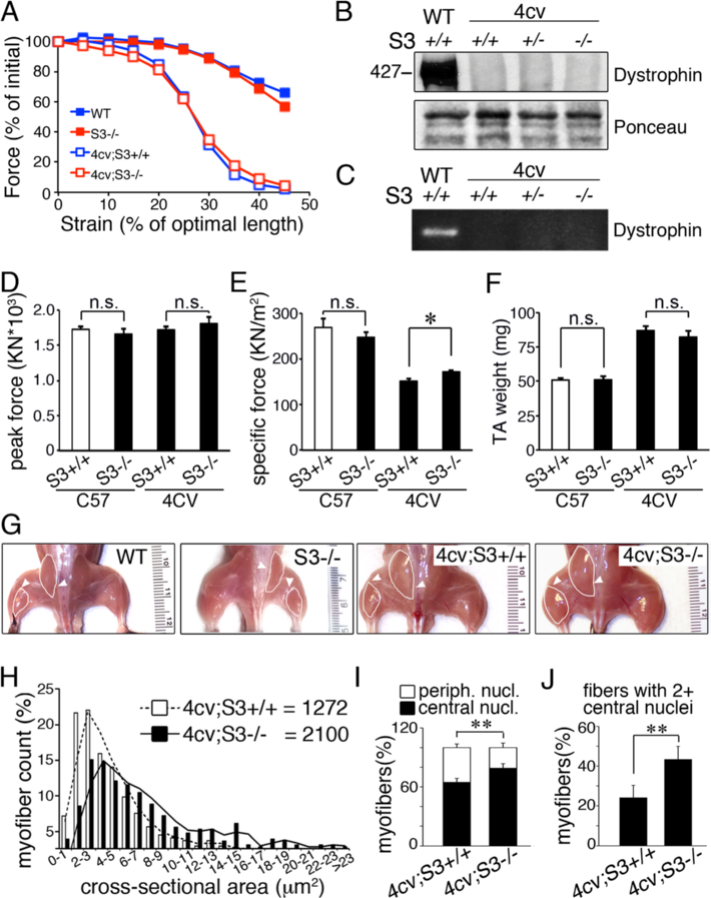
Syndecan-3 loss does not affect myofiber fragility in dystrophic muscle. **a** The TA muscles of non-dystrophic wild type mice (WT), non-dystrophic *Sdc3* ^*−/−*^ mice (S3*−/−*), and dystrophic mice either wild type for syndecan-3 (4cv;S3+/+) or syndecan-3 null (4cv;S3*−/−*) were assessed for contraction-induced muscle injuries. **b**, **c** Dystrophin protein levels (**b**, western blot) and gene expression (**c**, RT-PCR) are not restored in *mdx* ^*4cv*^ mice lacking syndecan-3. **d**, **e** Syndecan-3 loss modestly improves muscle force transduction in *mdx* ^*4cv*^ mice. Specific force (**e**) but not peak force (**d**) elicited by *mdx* ^*4cv*^ *;Sdc3* ^*−/−*^ TA muscles is increased compared to *mdx* ^*4cv*^ *;Sdc3* ^*+/+*^ TA muscles. Non-dystrophic *Sdc3* ^*+/+*^ and *Sdc3* ^*−/−*^ TA muscles showed no significant difference in peak or specific force and were averaged altogether and plotted as control (CTRL, *white bar* in **d** and **e**). **f**, **g** Syndecan-3 loss does not prevent compensatory hypertrophy and increased muscle mass in *mdx* ^*4cv*^ mice. Glutei and calves are highlighted in **g** and indicated by *white arrowheads*. **h** Myofiber cross-sectional area in *mdx* ^*4cv*^ *;Sdc3* ^*−/−*^ muscles (*white bars*) compared to *mdx* ^*4cv*^ *;Sdc3* ^*+/+*^ muscles (*black bars*). Inset numbers indicate the median myofiber cross-sectional area in square micrometers. **i**, **j** Increased myonuclear accretion in *mdx* ^*4cv*^ *;Sdc3* ^*−/−*^ muscles compared to *mdx* ^*4cv*^ *;Sdc3* ^*+/+*^ muscles. The fractions of centrally nucleated and peripherally nucleated myofibers were calculated and plotted for *mdx* ^*4cv*^ *;Sdc3* ^*−/−*^ and *mdx* ^*4cv*^ *;Sdc3* ^*+/+*^ muscles (**i**) and showed that the number of centrally nucleated myofibers containing two or more nuclei was increased (**j**). *Error bars* are S.E.M. ** = *p <* 0.01, * = *p <* 0.05, # = *p >* 0.05

An improvement in muscle histopathology and muscle function in dystrophic mice lacking syndecan-3 could result from reduced myofiber damage or from improved myofiber regeneration. Since syndecan-3 is not expressed in adult myofibers [32], reduced myofiber damage is unlikely to be responsible. In agreement with this, we found that contraction-induced injury was indistinguishable in syndecan-3 null dystrophic muscles compared to dystrophic muscles expressing syndecan-3 (Fig. 2a). No reversion of dystrophin expression (Fig. 2b, c) or compensatory overexpression of utrophin (data not shown) in *mdx* ^*4cv*^ *;Sdc3* ^*−/−*^ mice compared to *mdx* ^*4cv*^ *;Sdc3* ^*+/+*^ mice was observed. Consistently, peak force and specific force in *mdx* ^*4cv*^ *;Sdc3* ^*−/−*^ TA muscles were only slightly greater than the peak and specific force elicited by *mdx* ^*4cv*^ *;Sdc3* ^*+/+*^ TA muscles (Fig. 2d, e). Compensatory muscle hypertrophy is a hallmark of all *mdx* mouse strains, including the *mdx* ^*4cv*^ strain [39–43]. A whole-mouse examination of *mdx* ^*4cv*^ *;Sdc3* ^*−/−*^ mice showed only a modest increase in compensatory hypertrophy compared to *mdx* ^*4cv*^ *;Sdc3* ^*+/+*^ mice (Fig. 2g), consistent with the finding that the peak force is only modestly increased in *mdx* ^*4cv*^ *;Sdc3* ^*−/−*^ mice compared to *mdx* ^*4cv*^ *;Sdc3* ^*+/+*^ mice. Moreover, the TA average wet weight of *mdx* ^*4cv*^ *;Sdc3* ^*−/−*^ mice was not statistically different from the average wet TA weight of *mdx* ^*4cv*^ *;Sdc3* ^*+/+*^ mice (Fig. 2f).

Contraction-induced injury and muscle force measurement are carried out on individual muscles and represent a measure of muscle performance prior to damage. If syndecan-3 loss improved exercise performance by improving myofiber integrity, then both contraction-induced injury and muscle force would be improved. Instead, we find the opposite: neither contraction-induced injury nor muscle force are improved in *mdx* ^*4cv*^ *;Sdc3* ^*−/−*^ muscles. Therefore, loss of syndecan-3 in dystrophic muscle does not prevent myofiber rupture in response to stretch, yet the overall muscle histology is improved and is associated with an overall improvement in exercise performance. These apparently conflicting results could be explained if muscle regeneration was improved in *mdx* ^*4cv*^ *;Sdc3* ^*−/−*^ enhancing muscle function, improving fatigue resistance during exercise and reducing fibrosis.

We asked if enhanced regeneration in syndecan-3 null dystrophic muscle ameliorates the dystrophic phenotype. We observed an increase in myofiber area (Fig. 2h) accompanied by increases in centrally located nuclei (Fig. 2i) and numbers of myofibers with two or more centrally located nuclei (Fig. 2j) in *mdx* ^*4cv*^ *;Sdc3* ^*−/−*^ muscles compared to *mdx* ^*4cv*^ *;Sdc3* ^*+/+*^ muscles. These observations, together with our previous data that *Sdc3* ^*−/−*^ satellite cells generate larger myotubes ex vivo [14] support the hypothesis that syndecan-3 loss enhances myofiber regeneration in chronically injured muscles by increasing muscle progenitor contribution to damaged myofibers.

Fibro-adipogenic progenitors (FAPs) can convert to myogenic progenitors in a dystrophic environment [44]. To test whether myogenic conversion of FAPs was responsible for increased muscle regeneration and decreased fibrosis observed in *mdx* ^*4cv*^ *;Sdc3* ^*−/−*^ mice, we isolated FAPs from *mdx* ^*4cv*^ *;Sdc3* ^*+/+*^ and *mdx* ^*4cv*^ *;Sdc3* ^*−/−*^ mice and profiled them by qPCR for expression of myogenic markers. Although MyoD, Pax7, and Myf5 expression was detected in prospective satellite cells isolated from *mdx* ^*4cv*^ *;Sdc3* ^*+/+*^ or *mdx* ^*4cv*^ *;Sdc3* ^*−/−*^ muscle, no MyoD, Pax7, Pax3, and Myf5 expression was detected in FAPs isolated from either *mdx* ^*4cv*^ *;Sdc3* ^*+/+*^ or *mdx* ^*4cv*^ *;Sdc3* ^*−/−*^ muscle (Additional file 1: Figure S2). These results support the conclusion that in vivo conversion of FAPs to myogenic progenitors is negligible or absent in the *mdx* ^*4cv*^ dystrophic background and is not enhanced by syndecan-3 loss.

### Syndecan-3 loss improves regeneration repeatedly injured muscle and muscle aging

**Fig. 3:**
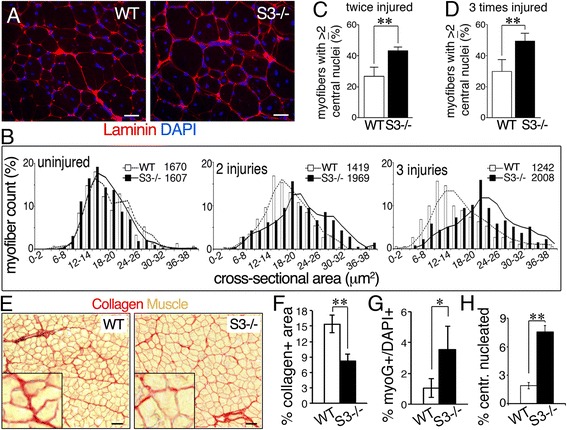
Syndecan-3 loss increases myofiber hypertrophy and myonuclear accretion in repeatedly injured muscles and improves muscle aging. **a** Wild type (WT) and *Sdc3* ^*−/−*^ (S3*−/−*) TA muscles harvested 3 weeks after three successive injuries and stained to detect laminin (*red*) and nuclei (*blue*). **b** Myofiber cross-sectional area of *Sdc3* ^*−/−*^ (S3*−/−*, *black bars*) and wild type (WT, *white bars*) from uninjured TA muscles and TA muscle injured with two or three successive BaCl_2_ injections (insets indicate median cross-sectional area in μm^2^). **c**, **d** Quantification of regenerating myofibers with centrally located nuclei after two (**c**) and three (**d**) successive injuries. **e** Fibrosis indicated by collagen staining is reduced in TA muscles from aged (2 years old) *Sdc3* ^*−/−*^ mice compared to TA muscles from age- and sex-matched wild type mice. **f** Quantification of (**e**). **g–h** Myogenin + cells (**g**) and the percent of centrally nucleated myofibers (**h**) in TA muscles from 2 years old *Sdc3* ^*−/−*^ mice are increased compared to TA muscles from age- and sex-matched wild type mice. *Scale bars* are 30 μm in **a** and 50 μm in **e**. *Error bars* are S.E.M. ** = *p <* 0.01; * = *p <* 0.05

Upon injury, depletion of Pax7+ satellite cells occurs in *Sdc3* ^*−/−*^ non-dystrophic muscle [14], similar to the Pax7+ satellite cell depletion observed in syndecan-3 null dystrophic muscle. To test whether the regenerative capacity of non-dystrophic *Sdc3* ^*−/−*^ muscle is similarly enhanced by syndecan-3 loss as is in dystrophic muscle, we repeatedly injured wild type and *Sdc3* ^*−/−*^ muscles and measured the extent of muscle regeneration. After three consecutive injuries, the median myofiber size of wild type muscle decreased, as expected to a loss of regenerative capacity (Fig. 3a, b). In contrast, the median myofiber size of repeatedly injured *Sdc3* ^*−/−*^ muscle progressively increased without an apparent increase in extracellular matrix deposition (Fig. 3a, b), similar to what was observed in syndecan-3 null dystrophic muscle (Fig. 2h). Moreover, the number of myofibers with more than two centrally located nuclei was increased in *Sdc3* ^*−/−*^ muscles that were injured either twice (Fig. 3c) or three times (Fig. 3d), a similar phenotype observed in *mdx* ^*4cv*^ *;Sdc3* ^*−/−*^ muscle compared to *mdx* ^*4cv*^ *;Sdc3* ^*+/+*^ muscle (Fig. 2j). Thus, lack of syndecan-3 appears to confer enhanced regenerative capacity in dystrophic muscle and in non-dystrophic muscle, repeatedly injured skeletal muscle.

During aging, a progressive loss of satellite cells occurs via loss of satellite cell self-renewal [15, 16, 45], which is thought to contribute to age-associated muscle fibrosis [21]. To determine if syndecan-3 loss affects fibrosis and muscle aging, we measured the levels of extracellular matrix deposition in 2-year-old wild type and *Sdc3* ^*−/−*^ muscles. Although an occasional accumulation of lipid droplets was previously described in aged *Sdc3* ^*−/−*^ muscle [12], a significant decrease in collagen was observed in aged *Sdc3* ^*−/−*^ muscle compared to aged wild type muscle (Fig. 3e, f). Reduced muscle fibrosis in old *Sdc3* ^*−/−*^ muscle was associated with increased numbers of myogenin + cells (Fig. 3g) and increased numbers of centrally nucleated fibers (Fig. 3h), suggesting that depletion of the pool of Pax7+ satellite cells upon activation in *Sdc3* ^*−/−*^ mice does not exhaust muscle regenerative capacity. Instead, syndecan-3 loss is associated with improved muscle in aged mice and improved regeneration in repeatedly injured muscle and in dystrophic muscle.

### Muscle progenitors distinct from satellite cells contribute minimally to muscle regeneration in the absence of syndecan-3

**Fig. 4:**
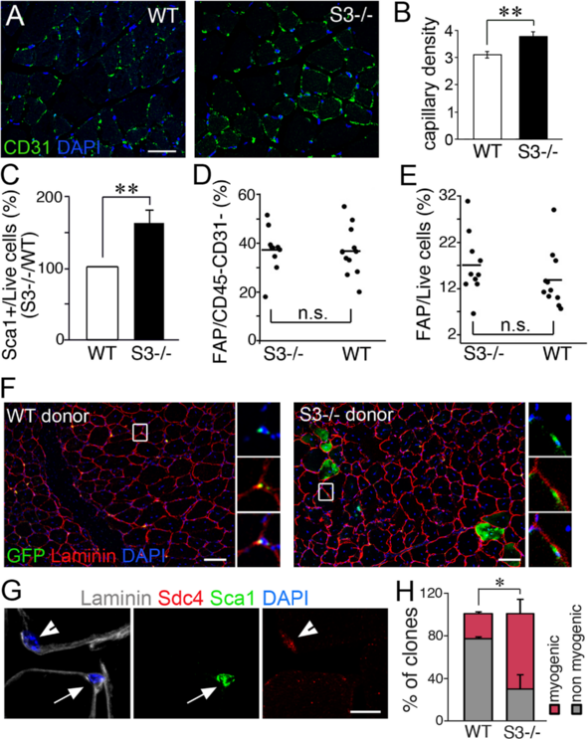
Vessel-associated progenitors unlikely contribute to myofibers in *Sdc3* ^*−/−*^ muscles. **a** Uninjured wild type (WT) and *Sdc3* ^*−/−*^ (S3*−/−*) TA muscles, immunostained to detect the endothelial cell marker CD31 (*green*) and nuclei (DAPI, *blue*) show an increase in capillary density in *Sdc3* ^*−/−*^ muscles compared to wild type muscles. Images representative of three biological replicates. **b** Quantification of **a. c** Flow cytometric analysis reveals increased numbers of Sca1+ cells in *Sdc3* ^*−/−*^ (S3*−/−*) muscles compared to wild type (WT) muscles. Gating scheme shown in Additional file 1: Figure S3A-D and F. **d**, **e** The numbers of fibro-adipogenic progenitors (FAPs) either in total (**d**) or expressed as percentage of all live cells (**e**) in uninjured muscle are comparable between wild type (WT) and syndecan-3 null (S3*−/−*) muscles. **f** FACS-isolated Sca1+ cells from wild type (WT) mice and *Sdc3* ^*−/−*^ (S3*−/−*) mice transplanted into wild type recipients that were injured with BaCl_2_ 4 h prior to transplant immunostained to detect GFP (*green*) and laminin (*red*) and stained with DAPI to detect nuclei 3 weeks post-transplantation. GFP staining is not entirely homogenous due to differences in tissue sectioning. Insets identify an interstitial WT donor cell (magnification, *left-hand side*) and a *Sdc3* ^*−/−*^ donor cell in the satellite cell niche (magnifications, *right-hand side*). **g** Wild type muscle cross sections immunostained to detect laminin (*white*), syndecan-4 (*red*), and Sca1 (*green*) and stained with DAPI to detect nuclei, show that syndecan-4 (Sdc4) is expressed by satellite cells and that Sca1+/Sdc4− cells are interstitial. **h** FACS-isolated Sca1+/Sdc4− cells from wild type (WT) and *Sdc3* ^*−/−*^ (S3*−/−*) muscles were cultured in myoblast growth medium at clonal density for 4 days and scored as myogenic (clones with myotubes) or non-myogenic clones. *Scale bars* are 100 μm in **a**, 30 μm in **f**, and 10 μm in **g**. *Error bars* indicate S.E.M. ** = *p <* 0.01

Muscle progenitor cells distinct from satellite cells may participate in muscle regeneration as illustrated by transplantation of pericytes [46], myoendothelial cells [47], and side population cells [48] into injured muscle. These muscle progenitor cells are associated with blood vessels [46–49]. *Sdc3* ^*−/−*^ muscle is more vascularized than wild type muscle as assessed by increased capillary density (Fig. 4a, b) and increased numbers of endothelial cells (Additional file 1: Figure S3A-E), consistent with the observation that syndecan-3 inhibits VEGF signaling in blood vessel development [50]. Thus, increased muscle vascularization associated with syndecan-3 loss may provide increased numbers of vessel-associated myogenic progenitors that could be responsible for the improvement in muscle maintenance and muscle regeneration occurring in *Sdc3* ^*−/−*^ muscle. Since these blood vessel-associated myogenic progenitors express Sca1, we assessed the percentage of Sca1+ cells present in the population of mononucleated cells in wild type and *Sdc3* ^*−/−*^ muscle. An increase in Sca1+ cells in *Sdc3* ^*−/−*^ muscle compared to wild type muscle is evident (Fig. 4c). Fibro-adipogenic progenitors (FAPs), which are not myogenic but also express Sca1, were not increased in *Sdc3* ^*−/−*^ muscle compared to wild type muscle (Fig. 4d, e). To test if any of these interstitial cell populations contributed to the enhanced regeneration we observe in *Sdc3* ^*−/−*^ muscle, we transplanted Sca1+/eGFP cells isolated from transgenic *ß-actin-eGFP;Sdc3* ^*+/+*^ and *ß-actin-eGFP;Sdc3* ^*−/−*^ muscle into injured wild type hosts (Additional file 1: Figure S3G). *Sdc3* ^*−/−*^ donor cells but not wild type donor cells engrafted the satellite cell niche and engrafted into myofibers (Fig. 4f). Although these data demonstrate that Sca1+ cells in *Sdc3* ^*−/−*^ mice contain myogenic progenitors capable of muscle engraftment upon transplantation, the extent of engraftment was minimal (observed only in two of five transplanted mice) and appears unlikely to account for the enhancement of muscle function and regeneration observed in dystrophic and non-dystrophic *Sdc3* ^*−/−*^ mice.

In addition to interstitial, non-myogenic cells, Sca1 is also expressed in a subpopulation of satellite cells marked by syndecan-4 [49, 51] and is induced upon satellite cell activation in a subpopulation of satellite cells that self-renew [52, 53]. Therefore, we asked if differences exist between wild type and *Sdc3* ^*−/−*^ muscle-derived Sca1+/Sdc4− cells (Fig. 4g). When Sca1+/Sdc4− cells from wild type and *Sdc3* ^*−/−*^ mice were isolated by FACS and cultured at clonal density, a higher percentage of myogenic Sca1+/Sdc4− clones was present in *Sdc3* ^*−/−*^ muscle as opposed to wild type muscle (Fig. 4h).

### Satellite cell homeostasis is altered in mice lacking syndecan-3

**Fig. 5:**
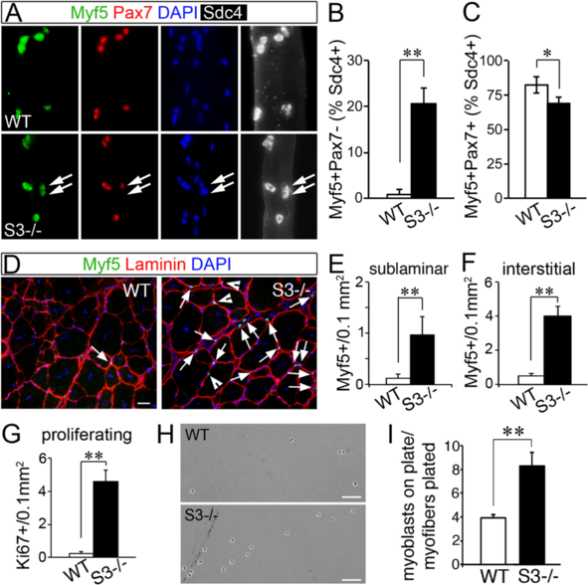
Syndecan-3 regulates myoblast homeostasis and migration. **a** Wild type (WT, *top panels*) and *Sdc3* ^*−/−*^ (S3*−/−*, *bottom panels*) myofibers cultured in suspension for 4 days, fixed, and immunostained to detect Sdc4 (*white*), Pax7 (*red*), Myf5 (*green*), and nuclei (blue). *Arrows* indicate a satellite cell doublet on a *Sdc3* ^*−/−*^ myofiber where one cell is Myf5 + Pax7+ and the other cell is Myf5 + Pax7−. **b**, **c** Quantification of Myf5 + Pax7− cells (**b**) and Myf5 + Pax7+ cells (**c**) as in **a**. **d** Muscle cross sections identifying Myf5+ cells (*green*) in *Sdc3* ^*−/−*^ (S3*−/−*) and wild type (WT) muscles 3 months post-injury. *Arrows* are interstitial cells; *arrowheads* are sublaminar cells. **e**, **f** Quantification of sublaminar (**e**) and interstitial (**f**) Myf5+ cells (normalized to area) in *Sdc3* ^*−/−*^ (S3*−/−*) and wild type (WT) muscles 3 months post-injury. **g** Quantification of the numbers of Ki67+ cells (normalized to area) in wild type (WT) and *Sdc3* ^*−/−*^ (S3*−/−*) muscles 3 months post-injury. **h** More myoblasts migrate away from *Sdc3* ^*−/−*^ (S3*−/−*) myofibers transferred to gelatin-coated coverslips after 2.5 days culture in suspension than from wild type (WT) myofibers. **i** Quantification of adherent myoblasts 4 h after myofiber transfer as in **g**. *Scale bars* are 100 μm in **a**, 50 μm in **e**, and 20 μm in **f**. *Error bars* are S.E.M. and ** = *p <* 0.01; * = *p <* 0.05

It appears unlikely that the restoration of regenerative capacity in syndecan-3 null dystrophic mice is due to non-satellite progenitors, since (i) we did not detect myogenic conversion of FAPs; and (ii) engraftment of transplanted Sca1+ cells from *Sdc3* ^*−/−*^ mice into muscle was only modestly increased compared to engraftment of wild type Sca1+ cells. Although the number of sublaminar Pax7+ cells is reduced in *Sdc3* ^*−/−*^ muscle following injury, the number of Sdc4+ satellite cells is paradoxically increased in *Sdc3* ^*−/−*^ muscle compared to wild type muscle. This could be explained by downregulation of Pax7 accompanied by an increase in myoblasts that express low levels of Pax7. To test these ideas, we first isolated myofibers and determined the relative immunreactivity for syndecan-4 (Sdc4), Myf5, and Pax7 in *Sdc3* ^*−/−*^ cells and in wild type cells (Fig. 5a–c). Nearly all Sdc4+ satellite cells on isolated myofibers were Myf5+ in both genotypes (Fig. 5a), confirming the validity of Sdc4 as a satellite cell marker. However, when we quantified Pax7+/Myf5+ cells and Pax7−/My5+ cells, few if any wild type cells were Pax7−/Myf5+ while nearly 25 % of *Sdc3* ^*−/−*^ Myf5+ cells were Pax7− (Fig. 5a–c), suggesting that Pax7 protein levels are lower in the absence of syndecan-3.

Since Pax7 promotes satellite cell quiescence [54, 55] and *Sdc3* ^*−/−*^ satellite cells are more prone to activation than wild type cells in uninjured muscle [14], it seems reasonable that reduced Pax7 levels (Fig. 5a–c) and loss of self-renewal capacity in *Sdc3* ^*−/−*^ satellite cells [14] are linked. If correct, then regenerated *Sdc3* ^*−/−*^ muscle, which is depleted of Pax7+ satellite cells, should possess activated satellite cells. As Myf5 protein is a marker for activated satellite cells, we assessed muscle sections of regenerated wild type and *Sdc3* ^*−/−*^ muscles for Myf5+ cells and found numerous Myf5+ sublaminar and interstitial cells in *Sdc3* ^*−/−*^ muscles that were not present in wild type muscles (Fig. 5d–f). The increase in Myf5+ cells was accompanied by an increase in Ki67+ cells (Fig. 5g), primarily localized to the interstitial space between myofibers. Remarkably, the total number of myogenic cells in regenerated *Sdc3* ^*−/−*^ muscle is twofold greater than the number of myogenic cells in wild type muscle (Table 1) and is consistent with our observation that a greater number of syndecan-4+ cells is present in uninjured *Sdc3* ^*−/−*^ muscle compared to wild type muscle [12]. Thus, after injury, satellite cells appear to redistribute in *Sdc3* ^*−/−*^ muscle, accompanied by a reduction in Pax7 protein levels and an expansion in Myf5+ cells, which are likely responsible for the increases in centrally located nuclei found in *Sdc3* ^*−/−*^ muscle and responsible for the enhanced regenerative capacity of *Sdc3* ^*−/−*^ muscle and *mdx* ^*4cv*^ *;Sdc3* ^*−/−*^ muscle.

**Table 1:** Total numbers of myogenic progenitors are increased in uninjured *Sdc3* ^*−/−*^ muscle compared to wild type muscle

	WT	S3*−/−*	Reference
Pax7 sublaminar (A)	3.62 ± 0.38	2.23 ± 0.14	Pisconti, JCB 2010
Myf5 sublaminar (B)	0.12 ± 0.08	0.97 ± 0.36	Fig. 5d, e
Total sublaminar	3.74	3.20	A + B
Myf5 interstitial (C)	0.5 ± 0.16	4.03 ± 0.57	Fig. 5d, f
Total myogenic cells	4.24	7.23	A + B + C

The numbers Pax7+ nuclei, sublaminar Myf5+ nuclei and interstitial Myf5+ nuclei normalized to area in the respective sections scored for wild type TA muscles (WT, column 2) and *Sdc3* ^*−/−*^ muscles (S3*−/−*, column 3) 3 months after an induced muscle injury. References refer to the source for scoring where statistical analysis can be found (column 4). The final row is a summation of each column with the total numbers of myogenic cells in the respective sections

Maintenance of interstitial Myf5+ cells in *Sdc3* ^*−/−*^ mice may reflect changes in *Sdc3* ^*−/−*^ cell adhesion since syndecans are adhesion molecules. *Sdc3* ^*−/−*^ myofiber-associated satellite cells appear less adhesive than wild type satellite cells (Additional file 1: Figure S4A-B). When isolated myofibers from wild type and *Sdc3* ^*−/−*^ muscles were cultured in suspension and then transferred onto gelatin-coated dishes, twofold more *Sdc3* ^*−/−*^ myoblasts adhered to the gelatin-coated surface than wild type myoblasts 4 h post-transfer (Fig. 5h, i and Additional file 1: Figure S4C). The propensity of *Sdc3* ^*−/−*^ satellite cells to migrate away from their native niche is consistent with the finding that the majority of Myf5+ and Ki67+ cells observed in regenerated *Sdc3* ^*−/−*^ muscle are located in the interstitial space, and supports the idea that the My5+ myoblast population observed in regenerated *Sdc3* ^*−/−*^ muscle is derived from satellite cells that migrated away from their niche.

## Discussion

In adult wild type muscle, Pax7+ satellite cells are quiescent and indispensable for muscle regeneration [56, 57]; Pax7 is necessary to maintain this population [58, 59]. Satellite cell niche components including Notch, syndecan-4, integrin-α7, Wnt, FGFs, HGF, the calcitonin receptor, and fibronectin play critical roles in maintaining satellite cells in their niche [12, 14, 15, 27, 60–64]. Syndecan-3, a transmembrane proteoglycan expressed in satellite cells and involved in regulating satellite cell responses to growth factors and to Notch [12, 14, 30], appears to promote satellite cell identity, the association of satellite cells with their niche and satellite cell quiescence.

**Fig. 6:**
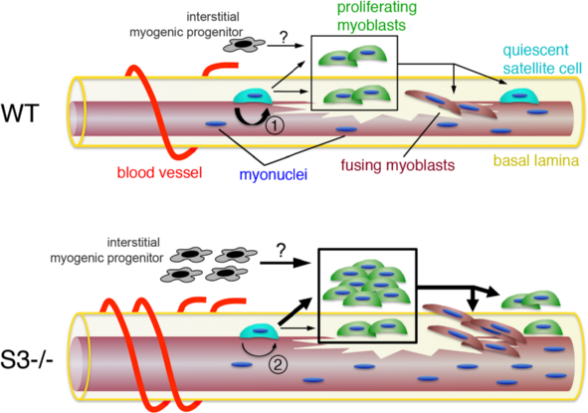
Syndecan-3 regulates satellite cell-niche interactions and satellite cell homeostasis. In wild type muscle (WT, *top drawing*) satellite cells (*light blue cells*) activate in response to myofiber injury and self-renew via asymmetric cell division (*1*) or proliferate as myoblasts (*green cells*) either underneath the basal lamina (*yellow*) or outside their niche, in the endomysium, and eventually differentiate (red mononucleated cells) to fuse to damaged myofibers or to one another. In *Sdc3* ^*−/−*^ muscle (S3*−/−*, *bottom drawing*), satellite cell self-renewal is decreased (*2*) leading to increased numbers of activated myoblasts, which proliferate mostly outside the niche, due to reduced adhesiveness to the myofiber. The increased number of proliferating myoblasts provides for increased numbers of differentiated myocytes that fuse to damaged myofibers leading to larger, hyperplastic regenerated myofibers. Since satellite cell self-renewal is decreased, a population of activated and proliferating myoblasts persists. Other myogenic progenitor cells distinct from satellite cells and possibly derived from blood vessel-associated progenitors (*gray cells*) may participate in muscle regeneration in *Sdc3* ^*−/−*^ muscles

Upon muscle injury, wild type satellite cells activate, rapidly induce the myogenic transcription factors MyoD and Myf5, and abandon their anatomical niche to migrate to the site of injury. These cells proliferate as myoblasts, eventually differentiating and fusing into damaged myofibers or with each other to form new myofibers [27, 29]. Compared to wild type satellite cells, Pax7 expression is reduced and Myf5 elevated in *Sdc3* ^*−/−*^ satellite cells at least 3 months post-injury. Thus, *Sdc3* ^*−/−*^ mice maintain Myf5 + Pax7− cells long term, which likely continue to proliferate, consistent with their reduction in Notch signaling [14], which, in turn promotes Pax7 expression and a return to quiescence [54, 65, 66]. The hypersensitivity of Syndecan-3 null satellite cells to HGF and FGF2 [12, 30] promotes satellite cell activation and proliferation [22, 67–69]. The appearance of an interstitial Myf5+ cell population is consistent with the reduced myofiber adherence and enhanced migration of *Sdc3* ^*−/−*^ satellite cells away from the myofiber, where the reduction in Notch signaling prevents re-homing to the satellite cell niche [70]. Thus, syndecan-3 loss leads to the sustained presence of increased numbers of proliferating myogenic progenitors, which provide for increased myofiber size and increased numbers of centrally nucleated myofibers (Fig. 6).

Since *Sdc3* ^*−/−*^ satellite cells proliferate slowly and show increased rates of cell death due to a defect in Notch signaling [14], the process of myonuclear accretion is slow and in the short lifespan of a mouse does not lead to an appreciable increase in muscle size. Nonetheless, a significant increase in satellite cell contribution to myofibers, shown by the presence of centrally nucleated myofibers, accompanied by a significant reduction in muscle fibrosis, is observed in wild type or dystrophic aged mice lacking syndecan-3. Thus, syndecan-3 loss appears to provide a lifelong benefit to muscle regenerative capacity in mice.

Although other potential myogenic progenitors, such as pericytes, myoendothelial cells, and side population cells, which are increased in *Sdc3* ^*−/−*^ muscle and show increased myogenicity in vitro, may contribute to interstitial and sublaminar myoblasts, the relative contribution of these cells appears low and may possibly be due to satellite cell contamination of the interstitial cell preparation. We cannot directly lineage trace the Myf5+ interstitial cells identified in regenerated *Sdc3* ^*−/−*^ muscle due to (i) the close proximity of Pax7 and syndecan-3 on the same chromosome, (ii) the lower levels of Pax7 in *Sdc3* ^*−/−*^ satellite cells, and (iii) the co-expression of MyoD and Myf5 by activated satellite cells and the interstitial myoblasts in *Sdc3* ^*−/−*^ muscle.

Loss of muscle regenerative capacity in the muscular dystrophies is often attributed to satellite cell exhaustion [3–9, 71]; however, there are only few experiments directly supporting this hypothesis. We utilized *mdx* ^*4cv*^ mice [37, 72], which develop a more severe form of muscular dystrophy than *mdx* mice that is exacerbated when challenged with exercise [42]. The dystrophy becomes more severe as the mice age, presumably due to the lower numbers of revertant fibers in *mdx* ^*4cv*^ mice than in *mdx* mice [38]. Loss of syndecan-3 in dystrophic mice reduces muscle fibrosis while improving exercise performance without ameliorating myofiber fragility or increasing the specific force. Since myofiber damage appears equivalent in dystrophic muscle with or without syndecan-3, we postulate that muscle regeneration is enhanced, leading to improved exercise performance. This conclusion is supported by the finding that *mdx* ^*4cv*^ *;Sdc3* ^*−/−*^ muscles contain more regenerating myofibers than *mdx* ^*4cv*^ *;Sdc3* ^*+/+*^ muscles and enhanced myonuclear accretion, consistent with a role for syndecan-3 in supporting Notch signals which promotes self-renewal while inhibiting myoblast fusion [14].

The *Sdc3* ^*−/−*^ satellite cell phenotypes appear cell autonomous as they occur in culture as well as in dystrophic mice lacking syndecan-3 and in aged *Sdc3* ^*−/−*^ mice. Overall the mechanism responsible for the enhancement of regeneration in double mutant *mdx* ^*4cv*^ *;Sdc3* ^*−/−*^ mice, the amelioration of the dystrophic phenotype, and the improvement of muscle maintenance in aged mice appears to be the failure of *Sdc3* ^*−/−*^ satellite cells to return to quiescence and re-home to their niche after activation, which maintains an expanding population of interstitial Myf5+ myoblasts. The numbers of *Sdc3* ^*−/−*^ myoblasts increase over time leading to an expanded muscle progenitor population in the muscle interstitium that eventually generates large, centrally nucleated myofibers (Fig. 6).

## Conclusions

*Sdc3* ^*−/−*^ mice maintain lifelong muscle regenerative capacity and resist injury-induced loss of regenerative capacity by maintaining a population of activated, Myf5+Pax7− satellite cells and a proliferating myoblast population in the myofiber interstitium. *Sdc3* ^*−/−*^ satellite cells do not appear exhausted in either dystrophic muscle or aged muscle apparently enhancing muscle regenerative capacity, identifying a new potential therapeutic target for the treatment and management of muscular dystrophies, repeated acute injuries and muscle aging.

## Additional file

Additional file 1: Supplementary figures. (PDF 4815 kb)

## References

[1] Cohn RD, Campbell KP (2000). Molecular basis of muscular dystrophies. Muscle Nerve.

[2] Straub V, Rafael JA, Chamberlain JS, Campbell KP (1997). Animal models for muscular dystrophy show different patterns of sarcolemmal disruption. J Cell Biol.

[3] Mouly V, Aamiri A, Bigot A, Cooper RN, Di Donna S, Furling D, Gidaro T, Jacquemin V, Mamchaoui K, Negroni E, Perie S, Renault V, Silva-Barbosa SD, Butler-Browne GS (2005). The mitotic clock in skeletal muscle regeneration, disease and cell mediated gene therapy. Acta Physiol Scand.

[4] Luz MAM, Marques MJ, Santo Neto H (2002). Impaired regeneration of dystrophin-deficient muscle fibers is caused by exhaustion of myogenic cells. Braz J Med Biol Res.

[5] Reimann J, Irintchev A, Wernig A (2000). Regenerative capacity and the number of satellite cells in soleus muscles of normal and mdx mice. Neuromuscul Disord.

[6] Maier F, Bornemann A (1999). Comparison of the muscle fiber diameter and satellite cell frequency in human muscle biopsies. Muscle Nerve.

[7] Decary S, Hamida CB, Mouly V, Barbet JP, Hentati F, Butler-Browne GS (2000). Shorter telomeres in dystrophic muscle consistent with extensive regeneration in young children. Neuromuscul Disord.

[8] Sacco A, Mourkioti F, Tran R, Choi J, Llewellyn M, Kraft P, Shkreli M, Delp S, Pomerantz JH, Artandi SE, Blau HM (2010). Short telomeres and stem cell exhaustion model Duchenne muscular dystrophy in mdx/mTR mice. Cell.

[9] Di Donna S, Renault V, Forestier C, Piron-Hamelin G, Thiesson D, Cooper RN, Ponsot E, Decary S, Amouri R, Hentati F, Butler-Browne GS, Mouly V (2000). Regenerative capacity of human satellite cells: the mitotic clock in cell transplantation. Neurol Sci.

[10] Dumont NA, Wang YX, von Maltzahn J, Pasut A, Bentzinger CF, Brun CE, Rudnicki MA (2015). Dystrophin expression in muscle stem cells regulates their polarity and asymmetric division. Nat Med.

[11] Bentzinger CF, Wang YX, von Maltzahn J, Soleimani VD, Yin H, Rudnicki MA (2013). Fibronectin regulates Wnt7a signaling and satellite cell expansion. Cell Stem Cell.

[12] Cornelison DDW, Wilcox-Adelman SA, Goetinck PF, Rauvala H, Rapraeger AC, Olwin BB (2004). Essential and separable roles for Syndecan-3 and Syndecan-4 in skeletal muscle development and regeneration. Genes Dev.

[13] Kuang S, Kuroda K, Le Grand F, Rudnicki MA (2007). Asymmetric self-renewal and commitment of satellite stem cells in muscle. Cell.

[14] Pisconti A, Cornelison DDW, Olguín HC, Antwine TL, Olwin BB (2010). Syndecan-3 and Notch cooperate in regulating adult myogenesis. J Cell Biol.

[15] Bernet JD, Doles JD, Hall JK, Kelly Tanaka K, Carter TA, Olwin BB (2014). p38 MAPK signaling underlies a cell-autonomous loss of stem cell self-renewal in skeletal muscle of aged mice. Nat Med.

[16] Cosgrove BD, Gilbert PM, Porpiglia E, Mourkioti F, Lee SP, Corbel SY, Llewellyn ME, Delp SL, Blau HM (2014). Rejuvenation of the muscle stem cell population restores strength to injured aged muscles. Nat Med.

[17] Carlson ME, Hsu M, Conboy IM. Imbalance between pSmad3 and Notch induces CDK inhibitors in old muscle stem cells. Nature. 2008 10.1038/nature07034 PMC7761661 18552838

[18] Sousa-Victor P, Gutarra S, García-Prat L, Rodriguez-Ubreva J, Ortet L, Ruiz-Bonilla V, Jardí M, Ballestar E, González S, Serrano AL, Perdiguero E, Muñoz-Cánoves P (2014). Geriatric muscle stem cells switch reversible quiescence into senescence. Nature.

[19] Price FD, von Maltzahn J, Bentzinger CF, Dumont NA, Yin H, Chang NC, Wilson DH, Frenette J, Rudnicki MA (2014). Inhibition of JAK-STAT signaling stimulates adult satellite cell function. Nat Med.

[20] García-Prat L, Martínez-Vicente M, Perdiguero E, Ortet L, Rodriguez-Ubreva J, Rebollo E, Ruiz-Bonilla V, Gutarra S, Ballestar E, Serrano AL, Sandri M, Muñoz-Cánoves P (2016). Autophagy maintains stemness by preventing senescence. Nature.

[21] Fry CS, Lee JD, Mula J, Kirby TJ, Jackson JR, Liu F, Yang L, Mendias CL, Dupont-Versteegden EE, McCarthy JJ, Peterson CA (2015). Inducible depletion of satellite cells in adult, sedentary mice impairs muscle regenerative capacity without affecting sarcopenia. Nat Med.

[22] Jones NC, Tyner KJ, Nibarger L, Stanley HM, Cornelison DDW, Fedorov YV, Olwin BB (2005). The p38alpha/beta MAPK functions as a molecular switch to activate the quiescent satellite cell. J Cell Biol.

[23] MAURO A (1961). Satellite cell of skeletal muscle fibers. J Biophys Biochem Cytol.

[24] Schultz E (1978). Changes in the satellite cells of growing muscle following denervation. Anat Rec.

[25] Palacios D, Mozzetta C, Consalvi S, Caretti G, Saccone V, Proserpio V, Marquez VE, Valente S, Mai A, Forcales SV, Sartorelli V, Puri PL (2010). TNF/p38α/polycomb signaling to Pax7 locus in satellite cells links inflammation to the epigenetic control of muscle regeneration. Cell Stem Cell.

[26] Crist CG, Montarras D, Buckingham M (2012). Muscle satellite cells are primed for myogenesis but maintain quiescence with sequestration of Myf5 mRNA targeted by microRNA-31 in mRNP granules. Cell Stem Cell.

[27] Olguín HC, Pisconti A (2012). Marking the tempo for myogenesis: Pax7 and the regulation of muscle stem cell fate decisions. J Cell Mol Med.

[28] Shea KL, Xiang W, LaPorta VS, Licht JD, Keller C, Basson MA, Brack AS (2010). Sprouty1 regulates reversible quiescence of a self-renewing adult muscle stem cell pool during regeneration. Cell Stem Cell.

[29] Siegel AL, Atchison K, Fisher KE, Davis GE, Cornelison DDW (2009). 3D timelapse analysis of muscle satellite cell motility. Stem Cells.

[30] Fuentealba L, Carey DJ, Brandan E (1999). Antisense inhibition of syndecan-3 expression during skeletal muscle differentiation accelerates myogenesis through a basic fibroblast growth factor-dependent mechanism. J Biol Chem.

[31] Casar JC, Cabello-Verrugio C, Olguin H, Aldunate R, Inestrosa NC, Brandan E (2004). Heparan sulfate proteoglycans are increased during skeletal muscle regeneration: requirement of syndecan-3 for successful fiber formation. J Cell Sci.

[32] Cornelison DD, Filla MS, Stanley HM, Rapraeger AC, Olwin BB (2001). Syndecan-3 and syndecan-4 specifically mark skeletal muscle satellite cells and are implicated in satellite cell maintenance and muscle regeneration. Dev Biol.

[33] Rapraeger AC (2000). Syndecan-regulated receptor signaling. J Cell Biol.

[34] Banks GB, Gregorevic P, Allen JM, Finn EE, Chamberlain JS (2007). Functional capacity of dystrophins carrying deletions in the N-terminal actin-binding domain. Hum Mol Genet.

[35] Caldwell CJ, Mattey DL, Weller RO (1990). Role of the basement membrane in the regeneration of skeletal muscle. Neuropathol Appl Neurobiol.

[36] Joe AWB, Yi L, Natarajan A, Le Grand F, So L, Wang J, Rudnicki MA, Rossi FMV (2010). Muscle injury activates resident fibro/adipogenic progenitors that facilitate myogenesis. Nat Cell Biol.

[37] Chapman VM, Miller DR, Armstrong D, Caskey CT (1989). Recovery of induced mutations for X chromosome-linked muscular dystrophy in mice. Proc Natl Acad Sci U S A.

[38] Danko I, Chapman V, Wolff JA (1992). The frequency of revertants in mdx mouse genetic models for Duchenne muscular dystrophy. Pediatr Res.

[39] Hakim CH, Duan D (2012). A marginal level of dystrophin partially ameliorates hindlimb muscle passive mechanical properties in dystrophin-null mice. Muscle Nerve.

[40] Li D, Shin J-H, Duan D (2011). iNOS ablation does not improve specific force of the extensor digitorum longus muscle in dystrophin-deficient mdx4cv mice. PLoS One.

[41] Hakim CH, Burkin DJ, Duan D (2013). Alpha 7 integrin preserves the function of the extensor digitorum longus muscle in dystrophin-null mice. J Appl Physiol.

[42] Zhang Y, Yue Y, Li L, Hakim CH, Zhang K, Thomas GD, Duan D (2013). Dual AAV therapy ameliorates exercise-induced muscle injury and functional ischemia in murine models of Duchenne muscular dystrophy. Hum Mol Genet.

[43] Banks GB, Combs AC, Odom GL, Bloch RJ, Chamberlain JS (2014). Muscle structure influences utrophin expression in mdx mice. PLoS Genet.

[44] Saccone V, Consalvi S, Giordani L, Mozzetta C, Barozzi I, Sandoná M, Ryan T, Rojas-Muñoz A, Madaro L, Fasanaro P, Borsellino G, De Bardi M, Frigè G, Termanini A, Sun X, Rossant J, Bruneau BG, Mercola M, Minucci S, Puri PL (2014). HDAC-regulated myomiRs control BAF60 variant exchange and direct the functional phenotype of fibro-adipogenic progenitors in dystrophic muscles. Genes Dev.

[45] Chakkalakal JV, Jones KM, Basson MA, Brack AS (2012). The aged niche disrupts muscle stem cell quiescence. Nature.

[46] Dellavalle A, Sampaolesi M, Tonlorenzi R, Tagliafico E, Sacchetti B, Perani L, Innocenzi A, Galvez BG, Messina G, Morosetti R, Li S, Belicchi M, Peretti G, Chamberlain JS, Wright WE, Torrente Y, Ferrari S, Bianco P, Cossu G (2007). Pericytes of human skeletal muscle are myogenic precursors distinct from satellite cells. Nat Cell Biol.

[47] Zheng B, Cao B, Crisan M, Sun B, Li G, Logar A, Yap S, Pollett JB, Drowley L, Cassino T, Gharaibeh B, Deasy BM, Huard J, Peault B (2007). Prospective identification of myogenic endothelial cells in human skeletal muscle. Nat Biotechnol.

[48] Asakura A, Rudnicki MA (2002). Side population cells from diverse adult tissues are capable of in vitro hematopoietic differentiation. Exp Hematol.

[49] Doyle MJ, Zhou S, Tanaka KK, Pisconti A, Farina NH, Sorrentino BP, Olwin BB (2011). Abcg2 labels multiple cell types in skeletal muscle and participates in muscle regeneration. J Cell Biol.

[50] De Rossi G, Whiteford JR (2013). A novel role for syndecan-3 in angiogenesis. F1000Res.

[51] Tanaka KK, Hall JK, Troy AA, Cornelison DDW, Majka SM, Olwin BB (2009). Syndecan-4-expressing muscle progenitor cells in the SP engraft as satellite cells during muscle regeneration. Cell Stem Cell.

[52] Troy A, Cadwallader A, Fedorov Y, Tyner K, Tanaka K, Olwin B. Coordination of satellite cell activation and self-renewal by par complex-dependent asymmetric activation of p38$\alpha$/$\beta$ MAPK. Cell Stem Cell. 2012. In press. 10.1016/j.stem.2012.05.025 PMC4077199 23040480

[53] Mitchell PO, Mills T, O'Connor RS, Kline ER, Graubert T, Dzierzak E, Pavlath GK (2005). Sca-1 negatively regulates proliferation and differentiation of muscle cells. Dev Biol.

[54] Bjornson CRR, Cheung TH, Liu L, Tripathi PV, Steeper KM, Rando TA (2012). Notch signaling is necessary to maintain quiescence in adult muscle stem cells. Stem Cells.

[55] Olguín HC, Olwin BB (2004). Pax-7 up-regulation inhibits myogenesis and cell cycle progression in satellite cells: a potential mechanism for self-renewal. Dev Biol.

[56] Murphy MM, Lawson JA, Mathew SJ, Hutcheson DA, Kardon G (2011). Satellite cells, connective tissue fibroblasts and their interactions are crucial for muscle regeneration. Development.

[57] Sambasivan R, Yao R, Kissenpfennig A, Van Wittenberghe L, Paldi AAS, Gayraud-Morel B, Guenou H, Malissen B, Tajbakhsh S, Galy A (2011). Pax7-expressing satellite cells are indispensable for adult skeletal muscle regeneration. Development.

[58] Günther S, Kim J, Kostin S, Lepper C, Fan C-M, Braun T (2013). Myf5-positive satellite cells contribute to Pax7-dependent long-term maintenance of adult muscle stem cells. Cell Stem Cell.

[59] von Maltzahn J, Jones AE, Parks RJ, Rudnicki MA (2013). Pax7 is critical for the normal function of satellite cells in adult skeletal muscle. Proc Natl Acad Sci U S A.

[60] Abou-Khalil R, Brack AS. Muscle stem cells and reversible quiescence: The role of sprouty. Cell Cycle. 2010;9 10.4161/cc.9.13.12149 20581433

[61] Kudla AJ, Jones NC, Rosenthal RS, Arthur K, Clase KL, Olwin BB (1998). The FGF receptor-1 tyrosine kinase domain regulates myogenesis but is not sufficient to stimulate proliferation. J Cell Biol.

[62] Le Grand F, Jones AE, Seale V, Scime A, Rudnicki MA (2009). Wnt7a activates the planar cell polarity pathway to drive the symmetric expansion of satellite stem cells. Cell Stem Cell.

[63] Polesskaya A, Seale P, Rudnicki MA (2003). Wnt signaling induces the myogenic specification of resident CD45+ adult stem cells during muscle regeneration. Cell.

[64] Sheehan SM, Tatsumi R, Temm-Grove CJ, Allen RE (2000). HGF is an autocrine growth factor for skeletal muscle satellite cells in vitro. Muscle Nerve.

[65] Fukada S-I, Yamaguchi M, Kokubo H, Ogawa R, Uezumi A, Yoneda T, Matev MM, Motohashi N, Ito T, Zolkiewska A, Johnson RL, Saga Y, Miyagoe-Suzuki Y, Tsujikawa K, Takeda S, Yamamoto H (2011). Hesr1 and Hesr3 are essential to generate undifferentiated quiescent satellite cells and to maintain satellite cell numbers. Development.

[66] Wen Y, Bi P, Liu W, Asakura A, Keller C, Kuang S (2012). Constitutive Notch activation upregulates Pax7 and promotes the self-renewal of skeletal muscle satellite cells. Mol Cell Biol.

[67] Jones NC, Fedorov YV, Rosenthal RS, Olwin BB (2001). ERK1/2 is required for myoblast proliferation but is dispensable for muscle gene expression and cell fusion. J Cell Physiol.

[68] Tatsumi R, Yamada M, Katsuki Y, Okamoto S, Ishizaki J, Mizunoya W, Ikeuchi Y, Hattori A, Shimokawa H, Allen RE (2006). Low-pH preparation of skeletal muscle satellite cells can be used to study activation in vitro. Int J Biochem Cell Biol.

[69] Johnson SE, Allen RE (1995). Activation of skeletal muscle satellite cells and the role of fibroblast growth factor receptors. Exp Cell Res.

[70] Bröhl D, Vasyutina E, Czajkowski MT, Griger J, Rassek C, Rahn H-P, Purfürst B, Wende H, Birchmeier C (2012). Colonization of the satellite cell niche by skeletal muscle progenitor cells depends on Notch signals. Dev Cell.

[71] Lund TC, Grange RW, Lowe DA (2007). Telomere shortening in diaphragm and tibialis anterior muscles of aged mdx mice. Muscle Nerve.

[72] Im WB, Phelps SF, Copen EH, Adams EG, Slightom JL, Chamberlain JS (1996). Differential expression of dystrophin isoforms in strains of mdx mice with different mutations. Hum Mol Genet.

