# Supplementary material for: Loss of niche-satellite cell interactions in syndecan-3 null mice alters muscle progenitor cell homeostasis improving muscle regeneration
Source: Skelet Muscle. 2016 Oct 4;6:34. doi: 10.1186/s13395-016-0104-8 (PMC5064903; doi:10.1186/s13395-016-0104-8)
Supplement: Additional file 1: — Supplementary figures. (PDF 4815 kb) [file 13395_2016_104_MOESM1_ESM.pdf]

SUPPLEMENTARY FIGURES

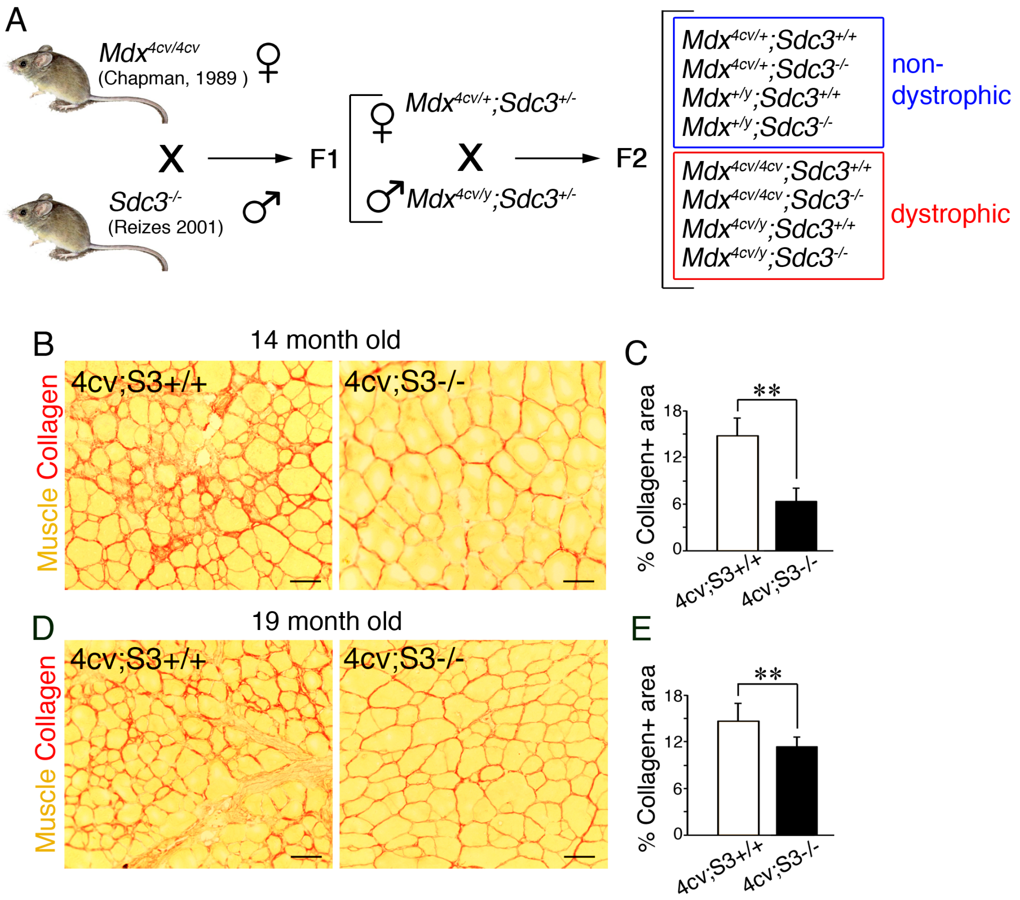

Figure S1: **A)** Schematic for the generation of double mutant mice lacking dystrophin and syndecan-3 expression. The double mutant colony was founded by two pairs of C57Bl/6 *Sdc3*$^{-/-}$ males bred to C57Bl/6 *mdx*$^{4cv/4cv}$ females, and maintained through several generations by breeding double heterozygous animals. Unless otherwise specified, *mdx*^{4cv} refers to either *mdx*$^{4cv/4cv}$ females or *mdx*$^{4cv/y}$ males. **B-E)** Improved muscle histopathology is maintained in dystrophic mice lacking syndecan-3 throughout life. TA muscles from 14 month old (B, C) and 19 month old (D, E) *mdx*^{4cv}*;Sdc3*$^{+/+}$ and *mdx*^{4cv}*;Sdc3*$^{-/-}$ mice were dissected, cryosectioned and stained with Sirius Red to detect muscle tissue (yellow) and collagen (red). The percentage of collagen+ area per unit area is reduced in *mdx*^{4cv} lacking syndecan-3. (C) and (E) are quantification of (B) and (D) from 3 mice, respectively. Scale bars are 30 μm in B and D. Error bars are S.E.M. **=p<0.01.

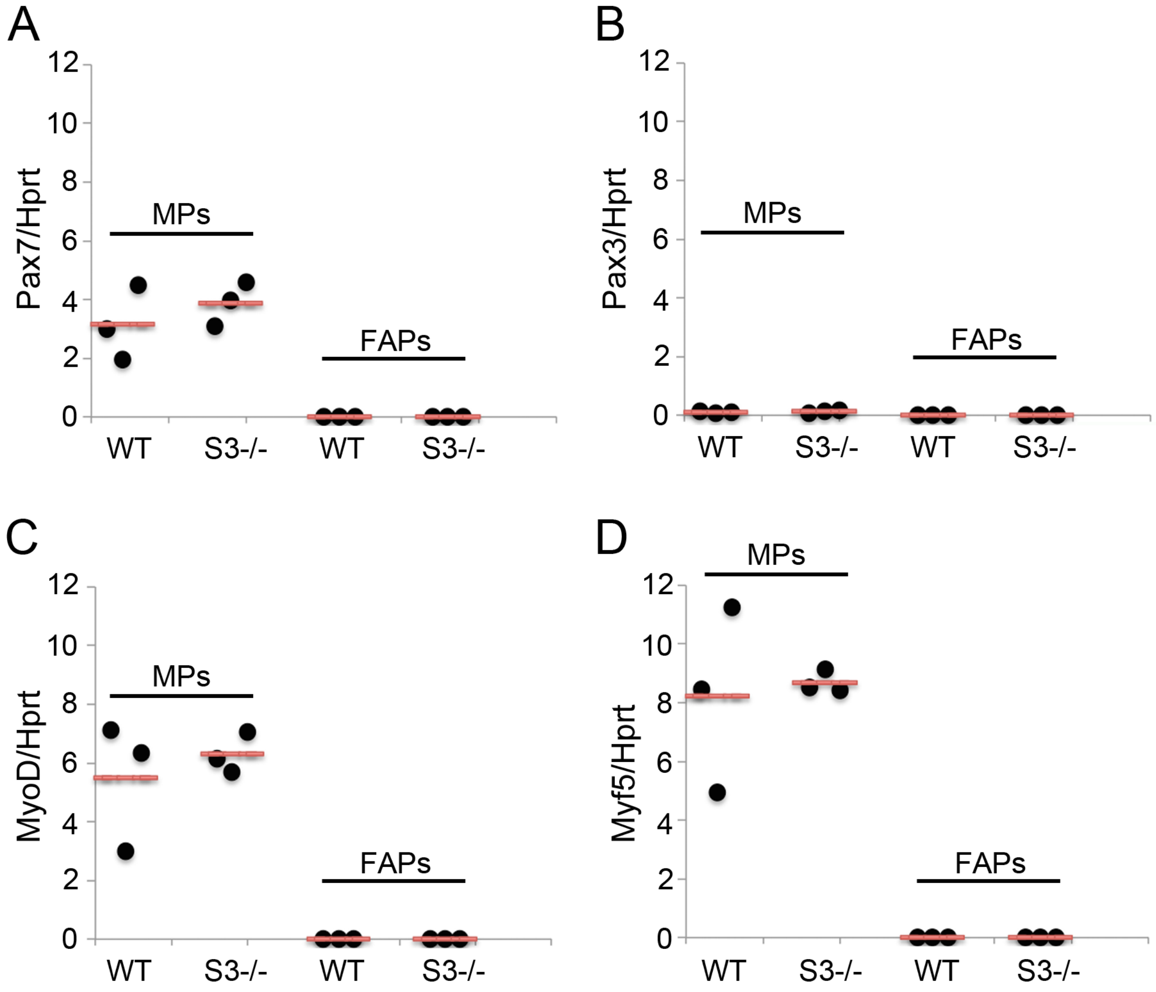

<u>Figure S2</u>: FAPs do not commit to the myogenic lineage in *mdx*^{4cv} mice, either in the presence or absence of syndecan-3. Fibro-adipogenic progenitors (FAPs) and muscle progenitors (MPs) were FACS-isolated from *mdx*^{4cv}*;Sdc3*$^{+/+}$ and *mdx*^{4cv}*;Sdc3*$^{-/-}$ muscles and profiled for their expression of myogenic markers: Pax7, Pax3, Myf5 and MyoD (normalized to Hprt levels). With the exception of Pax3, myogenic markers were expressed in MPs, while FAPs did not express any myogenic marker indicating that lack of syndecan-3 does not induce myogenic trans-differentiation in FAPs even in the presence of a dystrophic environment.

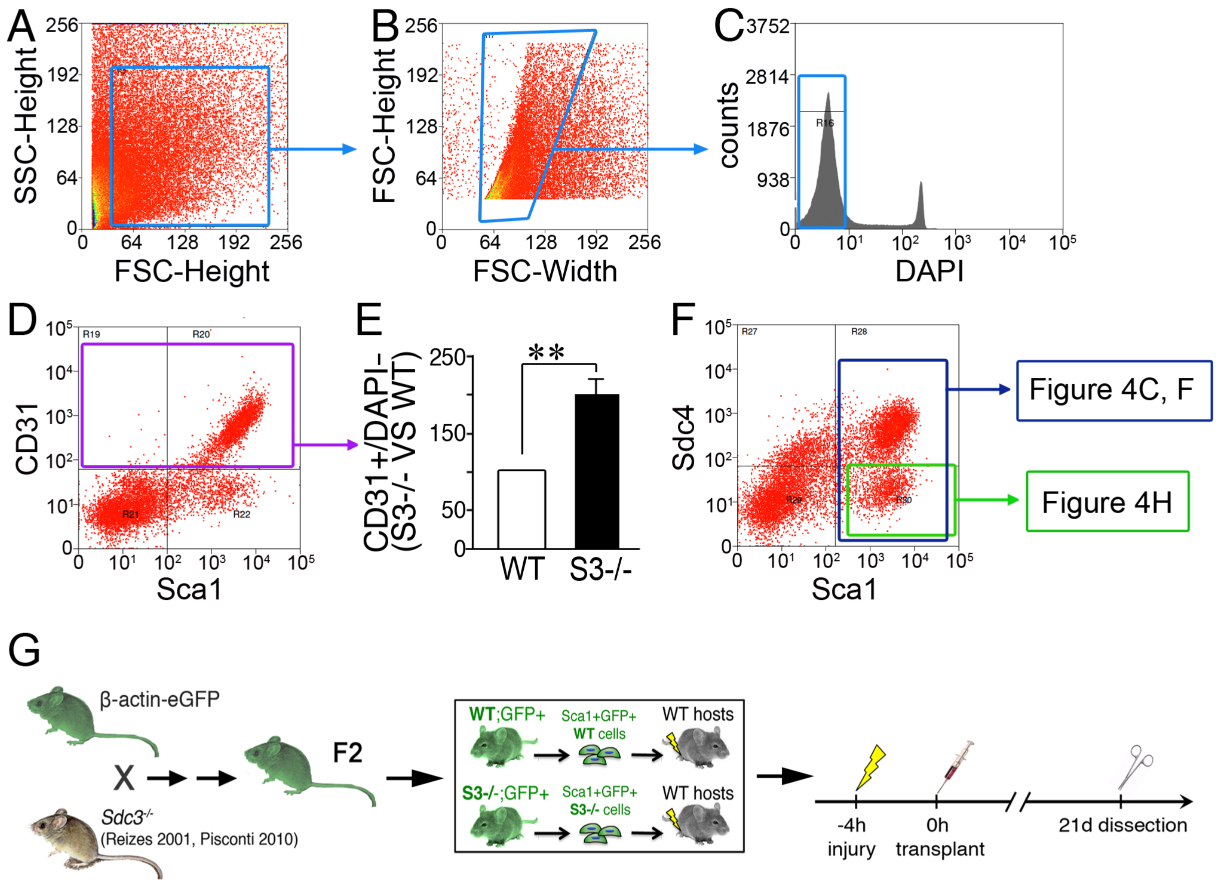

Figure S3: **A-F)** Flow cytometry gating scheme for profiling and quantitation of endothelial cells (D-E) and for FACS-isolation of Sca1+ and Sca1+Sdc4- cells (F). Total events in (A), SSC-Height vs FSC-Height, were selected based on forward and side scatter properties expected for satellite cells and proliferating myoblasts and then plotted as FSC-Height vs FSC-Width (B) to discriminate between single events and potential cell doublets or larger aggregates. Events that were bona fide single cells were selected and plotted as a function of DAPI staining intensity and gated for live cells impermeable to DAPI (C). Live cells were immunostained to identify (D) and quantify (E) Sca1+ CD31+ cells. Gating scheme for the FACS-isolation of total Sca1+ cells for quantitation of total Sca1+ cells (Figure 4C) and transplantation experiments (Fig. 4F), and of Sca1+Sdc4- cells for in vitro differentiation experiments (Fig. 4H). **G)** A schematic for the generation of *Sdc3*$^{-/-}$ mice expressing a transgenic eGFP under the control of a β-actin promoter.

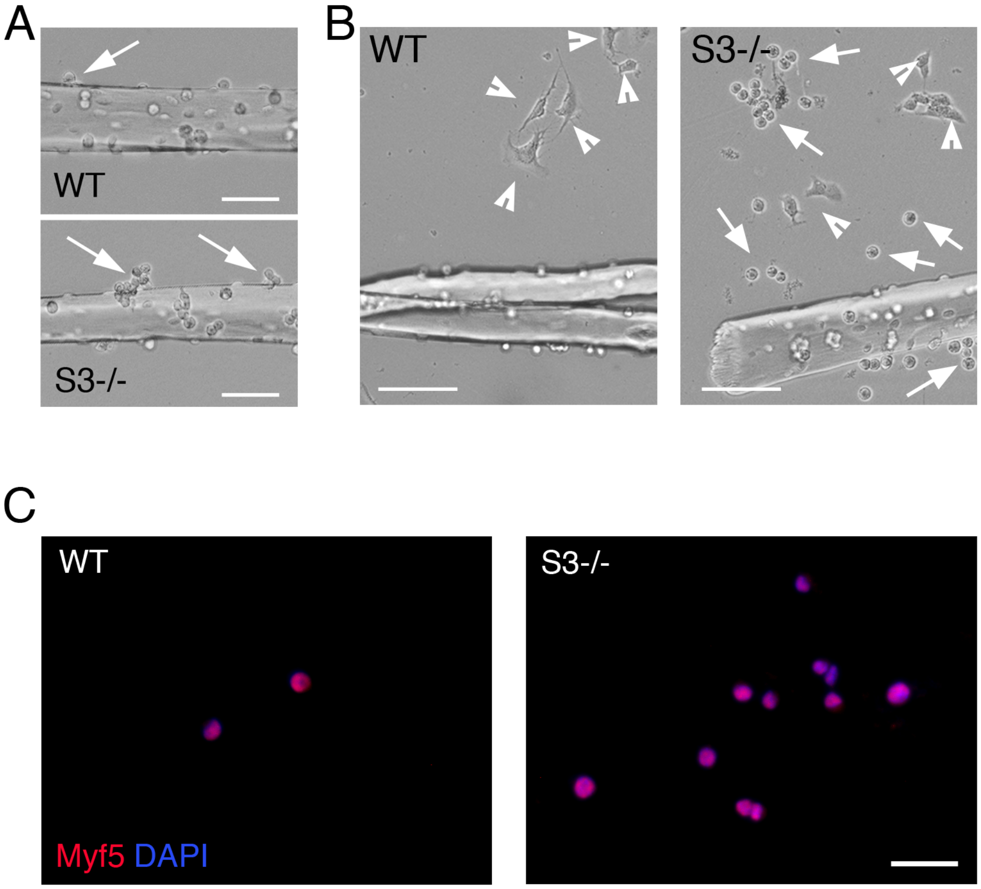

<u>Figure S4</u>: A) After 72h culture in suspension, *Sdc3*$^{-/-}$ myofibers show several groups of poorly adhesive satellite cells, which are rarely observed on wild type fibers. **B)** After a 72 h culture of wild type myofibers in suspension the majority of cells on the tissue culture surface are fibroblasts (arrowheads), while both fibroblasts and myoblasts (arrows) were detected attached to tissue culture plates of *Sdc3*$^{-/-}$ cultures. **C)** Cells that have migrated from wild type and *Sdc3*$^{-/-}$ (S3-/-) myofibres and adhered to the underlying gelatin-coated coverslip express Myf5. Representative images taken 4 hours after the myofibers were moved to gelatin-coated coverslips from suspension cultures are shown in (C), while a quantification of cell migration from the myofiber to the coverslip across three independent experiments is shown in Figure 5I. Scale bars are 100 µm A and B, 30 µm in C.

SUPPLEMENTARY METHODS

Mice

The double mutant *mdx*^{4cv}*;Sdc3*^{KO} mouse colony was established by breeding *mdx*$^{4cv/4cv}$ female founders to *Sdc3*$^{-/-}$ male founders, both lines sharing the same C57Bl/6 background (see supplemental Figure S1A for breeding program details). *Mdx*^{4cv}*;Sdc3*$^{-/-}$ and *mdx*^{4cv}*;Sdc3*$^{+/+}$ mice were examined across 4 subsequent generations (from F2 through F5) and no appreciable differences across generations within each genotype were observed. Unless otherwise indicated, mice between 4 and 6 months of age were used. *Sdc3*$^{+/+}$*;β-actin-eGFP* and *Sdc3*$^{-/-}$*;β-actin-eGFP* mice were generated and maintained at the University of Colorado, Boulder by crossing *Sdc3*$^{-/-}$ mice to *β-actin-eGFP* mice for two generations.
